# Decision-Support Tools Used in the Baltic Sea Area: Performance and End-User Preferences

**DOI:** 10.1007/s00267-020-01356-8

**Published:** 2020-09-10

**Authors:** Henrik Nygård, Floris M. van Beest, Lisa Bergqvist, Jacob Carstensen, Bo G. Gustafsson, Berit Hasler, Johanna Schumacher, Gerald Schernewski, Alexander Sokolov, Marianne Zandersen, Vivi Fleming

**Affiliations:** 1 Marine Research Centre, Finnish Environment Institute, Helsinki, Finland; 2 Department of Bioscience, Aarhus University, Roskilde, Denmark; 3 Baltic Sea Centre, Stockholm University, Stockholm, Sweden; 4 Tvärminne Zoological Station, University of Helsinki, Hanko, Finland; 5 Department of Environmental Science, Aarhus University, Roskilde, Denmark; 6 Leibniz Institute for Baltic Sea Research Warnemünde, Rostock, Germany; 7 Marine Research Institute, Klaipeda University, Klaipeda, Lithuania

**Keywords:** Marine management, Decision-making, Ecosystem approach, DAPSIWRM, Baltic Sea

## Abstract

Decision-support tools (DSTs) synthesize complex information to assist environmental managers in the decision-making process. Here, we review DSTs applied in the Baltic Sea area, to investigate how well the ecosystem approach is reflected in them, how different environmental problems are covered, and how well the tools meet the needs of the end users. The DSTs were evaluated based on (i) a set of performance criteria, (ii) information on end user preferences, (iii) how end users had been involved in tool development, and (iv) what experiences developers/hosts had on the use of the tools. We found that DSTs frequently addressed management needs related to eutrophication, biodiversity loss, or contaminant pollution. The majority of the DSTs addressed human activities, their pressures, or environmental status changes, but they seldom provided solutions for a complete ecosystem approach. In general, the DSTs were scientifically documented and transparent, but confidence in the outputs was poorly communicated. End user preferences were, apart from the shortcomings in communicating uncertainty, well accounted for in the DSTs. Although end users were commonly consulted during the DST development phase, they were not usually part of the development team. Answers from developers/hosts indicate that DSTs are not applied to their full potential. Deeper involvement of end users in the development phase could potentially increase the value and impact of DSTs. As a way forward, we propose streamlining the outputs of specific DSTs, so that they can be combined to a holistic insight of the consequences of management actions and serve the ecosystem approach in a better manner.

## Introduction

Coastal and marine ecosystems around the globe provide a range of services supporting the social and economic well-being of communities (Turner and Schaafsma 2015). The increasing use of marine resources leads to growing pressures and impacts on the marine environment, compromising the sustainable provisioning of ecosystem services (Airoldi and Beck 2007). In addition to sea-based activities, activities on land, e.g., agriculture and industry, also alter the condition of the marine environment through enhanced nutrient and pollutant runoff (HELCOM 2018a). Managing the use of resources, while sustaining ecological integrity, is a key challenge in marine environment management. This has led to the development of ecosystem-based management, striving to ensure that marine ecosystems are well functioning and able to support sustainable delivery of ecosystem goods and services (Elliott 2011; Borja et al. 2016).

The DAPSI(W)R(M) concept (Elliott et al. 2017), later in this paper referred to as DAPSIWRM, provides a general structuring and holistic framework for use in environmental management. The concept has evolved over many years, rooting back to the PSR framework that was proposed by Rapport and Friend (1979), further adapted by several international organizations (OECD 1993; EPA 1994; UNEP 1994) and expanded to DPSIR (EEA 1999), before stepwise transforming into its current format (Elliott et al. 2017).

The DAPSIWRM concept describes links and interactions between drivers (defining the needs), activities (human activities to fill the needs), pressures (caused by the activities), state changes (how the pressures affect the environment), impacts on welfare (how society is impacted), and response using measures (management actions). The ability of the DAPSIWRM framework to link natural and societal information facilitates integrated marine management while incorporating the ecosystem approach (Elliott et al. 2017). Ecosystem-based management relies on information of various components from the marine environment and on the sources and magnitude of pressures that impact marine ecosystem services. Consequently, vast amounts of information need to be synthesized to make well-informed decisions (Elliott 2013; Scharin et al. 2016).

Decision-support tools (DSTs) combine, process and condense large amounts of information, assisting the manager in the decision-making process. Decision makers at local, national, and intergovernmental level need this information to, for example, evaluate the current status of the marine environment, and estimate the most cost-efficient measures that are needed to restore impaired status (Balana et al. 2011) or to facilitate spatial planning (e.g., Bagstad et al. 2013, Stelzenmüller et al. 2013). DSTs allow a structured process where alternative options can be compared, improving the transparency of the decision-making process (Nicholson et al. 2020; Sullivan 2004; Ward 2007). Technically, DSTs are expected to produce outcomes, as realistic as possible, that answer a specific set of questions; for the eutrophication problem, as an example, questions such as: what is the eutrophication status of a sea area? How much nutrient pressure can it tolerate? What are the benefits of nutrient abatement? What are the most cost-efficient management actions?

The marine environment is protected by a large number of international and national legislation and policies (Boyes and Elliott 2014; Reusch et al. 2018). In the Baltic Sea, environmental problems and subsequent socio-economic impacts are addressed through regional, European and global policies and agreements, directed toward sustainable management of the Baltic Sea. The HELCOM Baltic Sea Action Plan (BSAP) is an example of an international regional agreement. On the European level, many environmental EU directives (Marine Strategy Framework Directive (MSFD), Water Framework Directive (WFD), Habitats Directive, Waste Framework Directive, Maritime Spatial Planning Directive) as well as sectoral policies (e.g., Integrated Maritime Policy, Common Fisheries Policy, Common Agricultural Policy) and strategies (Strategy on Adaptation to Climate Change, Biodiversity Strategy) exist. The implementation of these initiatives challenges the coastal states, that strive, both individually and collectively, to assess the potential outcomes of choices and decisions regarding the management of human actions and their impact on the environment. These decisions are guided by the policy requirements but they need to be based on first-hand information from the environment and the society. This is where DSTs are essential. The DSTs developed and applied in the Baltic Sea and its drainage basin range from quantitative assessment tools to model-based or operational systems linked to databases, but also include tools that handle more descriptive, nonquantitative information.

Here, we provide an overview of DSTs developed for application in environmental management of the Baltic Sea and its drainage area. First, we made an inventory, where the DSTs are categorized according to the DAPSIWRM framework and the environmental problems they address. Particularly, the role of the DSTs within the DAPSIWRM context, in terms of how well its components and linkages are covered, was investigated. Second, we developed a framework to assess DST performance and to understand their strengths and weaknesses. Third, we assessed the DSTs against the end user needs. Finally, we investigated the development and maintenance of the DSTs, addressing questions such as how end users had been involved in the tool development and how well the sustainability of the DSTs were ensured.

## Material and Methods

### Definition of DST

DSTs can be defined very broadly to include any tool that condenses complex information into an easier understandable format to be applied in decision-making (Sullivan 2004). Such tools include, for example quantitative assessment tools, model-based management tools and operational systems linked to databases. To structure our overview and to have a common understanding of the term “DST”, with focus on marine environmental management in the Baltic Sea, a definition for use in the BONUS DESTONY project^1^ was elaborated and a set of five definition criteria (DC) were formulated (Table 1). Ideally, a DESTONY DST should fulfill all these criteria, but in practice, they turned out to be very demanding. To include a broader range of DSTs in the inventory, fulfilling all DC was not considered as a requirement. However, this implies that we do not cover all tools (e.g., models) fulfilling few of the DC. In the performance analyses, we included only DSTs fulfilling more than four of the DC, to ensure comparability.

**Table 1:** DESTONY DST definition criteria (DC)

#	Definition criteria (DC)
(1)	The tool is interactive in the sense that the end user is requested for input data or information and will subsequently get outputs related to that. If the tool is based on a non-dynamic model that cannot show different outcomes, the tool is not considered interactive.
(2)	The tool is virtual in the sense that it can be accessed and operated through the internet. A tool is not virtual if you need to download it to your computer.
(3)	The purpose of the tool is to support decision-making in relation to degradation of the aquatic environment at local, regional, national, or international management scale. A single indicator is not seen as a tool.
(4)	The tool is primarily developed for use in the Baltic Sea or its drainage basin. If the tool is originally developed for other sea areas but adapted primarily to the Baltic Sea the criteria applies. If the tool is restricted to national waters, it needs to cover any of the DESTONY participating countries (Finland, Sweden, Germany, Denmark).
(5)	The tool is applicable and accessible by the end user (whether policy maker or expert involved in management) without unreasonable effort. The criteria does not apply in case of unreasonable efforts such as the tool cannot be found, or the tool needs to be used by the host.

### Inventory

As a basis for the inventory of available DSTs intended for the Baltic Sea and its drainage area, environmental management problems, where decision-making is needed, were identified (e.g., eutrophication, contaminant pollution, loss of biodiversity, etc.). Responsibility for reviewing the availability of DST within these problem areas was then distributed among the project participants based on specific expertise. As participants in the project consortium have been widely involved in both developing and using DSTs, there was a good understanding of the availability of DST already from the start. In addition, web searches were performed to ensure that other tools were not overlooked. For problem areas where there was no expertise in the project consortium (e.g., nonindigenous species, underwater noise, litter), interviews with experts in the field were made in order to identify DSTs. Ongoing and finished BONUS projects^2^ were also contacted to scan for recently developed tools or tools still under development. The list of identified tools was sent as part of the questionnaire to end users and stakeholders and they were asked to add potential DSTs missed.

Information about the DSTs and their performance were collected using a common template. The template consisted of three parts: (1) general information including problems, policies and DAPSIWRM components addressed, platform used, type of input data needed, description of outputs, a short description and information on developer/host, and where to find the tool as well as links to specific examples where the tool had been used; (2) DC (see Table 1); and (3) fifteen performance criteria (PC) (see Table 2). The latter was described both in words and with a 1–5 scale to further evaluate the tools. The filled templates (excluding the performance scoring) were sent to the developers or hosts of the DSTs for cross-checking and commenting, and the templates were updated accordingly. However, responses from hosts were received for only two thirds of the DTSs.

**Table 2:** The performance criteria (PC) and scoring classes (1–5) used to evaluate the tools

Definition of the performance criteria (PC)	Evaluation scale
PC1: Scientific documentation	
Has the DST been documented in scientific publication, etc.?	1 = no documentation found
	2 = earlier version in web but outdated
	3 = earlier version in report or scientific paper but outdated
	4 = updated documentation in web
	5 = updated documentation in report and/or scientific paper
PC2: Complexity of method	
How simple or complex is the method used for calculating the output?	1 = no quantitative analysis is applied; method is qualitative
	2 = simple quantitative method or descriptive statistics, e.g., one-out-all-out, sum, average, median
	3 = fairly simple quantitative statistics, e.g., weighted average, regression
	4 = complex quantitative methods, e.g., multidimensional statistics
	5 = very complex analysis, e.g., dynamic models or combinations of several tools and processes
PC3: Transparency of the DST	
Are all the processing described, is the code public, documentation, understandable? Are underlying methods/calculations transparent for the user?	1 = no description of processes
	2 = basic idea of the DST is explained but not in detail
	3 = basic idea explained and some metaproducts can be viewed
	4 = process is described in detail and all steps can be viewed
	5 = process is described in such detail that it could be repeated, code/tool may be viewed, all steps can be obtained
PC4: Management relevance to the Baltic Sea	
To what extent is the output related to making decisions on responses/measures?	1 = not directly related to decision-making
	2 = output is related to questions that require decision-making
	3 = output can be processed further to support decision-making
	4 = output can easily be combined with other information to support decision-making
	5 = output is directly supporting decision-making
PC5: Spatial limitations	
Is the spatial scale of the tool restricted or can it be adapted according to management needs (e.g., applied on a local as well as national level)?	1 = permanently fixed spatially
	2 = spatially fixed, and could be changed only through excessive reconfiguration of the tool
	3 = spatially fixed, but could be extended through relatively simple adjustments to the tool
	4 = there is a couple of alternative spatial options or some flexibility, but not unlimited possibilities
	5 = no spatial restrictions, DST can be adapted according to management needs
PC6: Temporal limitations	
Is the tool dynamic, i.e., describing changes over time? Does the output have a temporal dimension that can be expressed as years?	1 = output has no temporal dimension
	2 = a temporal dimension can easily be achieved through repetition
	3 = output shows results between two points in time
	4 = output shows results between several points in time
	5 = output extends over time, to the extent that it can express detailed changes resulting to management responses
PC7: Confidence assessment of results/level of uncertainty	
Does the tool assess the uncertainty associated with the output, and does this assessment account for all or a subset of potential uncertainties?	1 = no confidence expressed, or confidence expressed only for meta-products but not the end product. Uncertainty assessed only using alternative scenario modeling, sensitivity analyses, or expected outcomes of different scenarios
	2 = simple confidence criteria, e.g., qualitative expert judgment
	3 = non-comprehensive confidence criteria, covers only one or two aspects (spatio-temporal, methodological, or confidence-of-classification)
	4 = multifaceted confidence assessment partly relying on expert judgment, including spatio-temporal, methodological, and confidence-of-classification
	5 = completely data-driven multifaceted confidence assessment, including spatio-temporal, methodological, and confidence-of-classification
PC8: Data dependencies	
Does the tool work with missing values? Is it sensitive to changes in the type of input? Quantitative/qualitative data?	1 = can use only one type of information (whether qualitative or quantitative), very sensitive to missing values
	2 = can handle only one type of information or strong restrictions to the format or type of input data, but can handle missing values
	3 = flexible to different types of input data but with some restrictions, can deal with only qualitative or quantitative information, can handle missing values
	4 = no restrictions to the type of input data, can handle missing values, but can deal with only qualitative or quantitative information
	5 = input data can be qualitative/quantitative, is not sensitive to different types of input data, can handle missing values
PC9: Testing and validation	
Has the DST been applied to different systems and tested independently?	1 = no testing involved
	2 = has been tested/applied once
	3 = has been tested/applied in several cases but in a limited number of systems
	4 = has been tested/applied in several contexts
	5 = has been applied to several cases in different types of contexts, and tested thoroughly
PC10: Transferability	
How easily can the tool be adapted to other systems (e.g., North Sea, fresh water systems, etc.) by the end user?	1 = not applicable to other systems
	2 = applying to other systems would require reconstruction
	3 = applying to other systems would require considerable updates
	4 = can be applied to other systems with minor adjustments
	5 = can be directly applied to other systems
PC11: Thematic broadness	
How generic is the DST? For example, which and how many policy issues (e.g., eutrophication, biodiversity, pollution, maritime activities etc.) does it address?	1 = the DST is specific to an environmental policy issue (e.g., eutrophication) and highly specific to its aspects; it addresses e.g., a specific species, habitat, nutrient levels, etc.
	2 = the DST is specific to an environmental policy issue (e.g., eutrophication) but can address different aspects of it (e.g., indirect and direct effects)
	3 = a single application of the tool can deal with different environmental policy issues, but only one at a time (e.g., it can be applied to biodiversity or eutrophication, but not simultaneously)
	4 = the DST addresses two environmental policy issue at once (e.g., eutrophication and biodiversity)
	5 = the DST is highly flexible and can address various environmental policy issues at once
PC12: Broadness of components of the DPSIR/DAPSIWRM addressed	
How broadly does the tool handle the management chain of events, from drivers to pressures, state changes, impacts to environment, social impacts, and responses of society (e.g., components in the DPSIR/DAPSIWRM cycle?) How many components does it address?	1 = very narrow use, restricted to one or few interactions
	2 = narrow, covers only one segment in the DAPSIWRM cycle, and inspects it narrowly
	3 = covers only one segment in the DAPSIWRM cycle, but inspects it broadly
	4 = covers two segments in the DAPSIWRM cycle
	5 = very generic, covering three or more segments in the DAPSIWRM cycle broadly
PC13: Suitability to components operationally applied in the Baltic Sea	
How well does the tool fit in with the approaches and methodology already agreed upon in the area? Are the existing operational input components, e.g., monitoring data, indicators, compatible with the tool when applied in the Baltic Sea? Or should the input data be created/collected separately. Is the output directly suitable as input, or collaborative interpretation with output from other operational tools?	1 = tool is not compatible to operational input or output components
	2 = tool is not fully compatible to operational input and output components, but could be applied with some adjustments
	3 = tool is not fully compatible to input components, but output can be applied operationally
	4 = tool is fully compatible to input components applied in the Baltic Sea, but output requires further adjustments
	5 = both input and output components are applied operationally in the Baltic Sea
PC14: Ease of use/expertise required	
Is the tool generally applicable to non-expert users or restricted to experts? Is the DST easy to apply? Is there need for expertise in a specific field (e.g., marine ecology, economics, policy, etc.)?	1 = can be applied only by dedicated experts throughout the process
	2 = tool is applied by experts, but less experienced users can interact during selected phases
	3 = application of the tool requires participation in a special training course
	4 = anyone can apply the tool after extensive reading of the manual
	5 = the tool is easy to use, and no special expertise are required
PC15: Time effort	
How much time is needed to apply the DST? i.e., How much time is needed from the choice of the tool for a specific problem to the output of concrete/usable results?	1 = both preparation and application of the DST are time consuming (weeks or months)
	2 = preparation of the tool is rather quick (days), but application is time consuming (weeks or months)
	3 = preparation of the application is time consuming (weeks or months), but the application is rather quick (days)
	4 = both preparation and application of the DST are rather quick (days)
	5 = the DST can be directly applied and provides immediate results (e.g., in (stakeholder) meetings) (within hours/one day)

### Performance Criteria (PC)

A set of fifteen PC was defined to evaluate in greater detail how the tools work and how they differ from each other (see Table 2). The PC mirror the scientific foundation of the tools, how well they fit into Baltic Sea management as well as their user-friendliness, reflecting characteristics such as quality and relevance, that can be ranked according to general performance, and descriptive qualities that need to be considered case-specifically in the ranking. The aim was not to rank the tools based on the PC but rather to shed light on the applicability and transparency of the tools. For each criterion, a five-step scale was developed to reflect how well the tools respond to the criteria. The scale is categorical, but with increasing relevance. Generally, the value of 1 refers to “not fulfilling the criterion” whereas 5 refers to “fully complying with the criterion”. The scale is defined separately for each criterion.

### End User Involvement and Use of DSTs

As the filled DST templates were sent to the hosts for cross-checking, a questionnaire regarding the development of the tool and involvement of end users was attached. This questionnaire aimed at gaining understanding of why and how the tool was originally developed, if end users were involved in the development, and if the hosts were satisfied with how the tool had been applied in environmental management. The questionnaire was structured according to five themes, each with a set of questions/statements to be answered with yes or no: (1) initiation of tool development; including questions on what motivated the development of the tool, (2) defining end users; to find out if and at what stage in the development phase end users were identified, (3) end-user involvement; with questions on how end users were involved in the development process, (4) maintaining and updating; to find out if and by whom the tool is maintained and updated, and (5) use of the tool; asking if the host is satisfied with the way the tool has been used. The full questionnaire can be found in Online Resource 1. The survey was answered by 27 DST hosts out of 42.

Based on the results from the questionnaire on end-user involvement in development and use of DSTs, we examined if there are performance differences between (a) tools involving end users in the development phase, (b) tools that have been developed as a direct response to a management need, (c) tools where developers/hosts are satisfied with how tools have been used, and (d) tools where this has not been the case.

### End User Preferences

An online questionnaire was created to assess end users’ level of knowledge about DSTs, their own use of the tools and their preferences for the predefined PC (not including the scoring categories). End users were asked for their perception of importance of the DST PC into four categories: (1) very important, (2) important, (3) not so important, (4) not important at all. The questionnaire was distributed using the online software JotForm and made available during March 2019 (the questionnaire can be found in Online Resource 2). Identified end users of DSTs included representatives of national, regional, and local administrations, international organizations and institutions (e.g., HELCOM, VASAB, ICES, EU), nongovernmental organizations (NGOs), as well as researchers represented in national or international working groups dealing with management issues related to the environment of the Baltic Sea and its drainage basin. In total, 811 email invitations were sent to potential DST end users in all Baltic Sea countries. In addition, an invitation to participate was launched on the webpage and social media pages (Twitter and Facebook) of the Stockholm University Baltic Sea Center.

In total, 108 responses were received. Number of responses varied strongly between countries. 45% of all participants indicated to be working in Sweden, 19% in Finland, and 9% in Germany. Lithuania and Estonia were represented by 6%, Denmark and Latvia by 4%, and Poland and Russia by 2%. Three participants (3%) stated that they are working in multiple countries and one EU representative from Ireland participated. Around half of the participants (53%) were representatives of public administration, 38% from research institutes and universities, and 8% from NGOs. Others included participants from consultancies and private companies and made up 5%. Participants had diverse thematic backgrounds, such as eutrophication (55%), marine spatial planning (MSP) (38%), marine habitats (36%), nature protection (32%), marine litter (28%), hazardous substances (26%), fisheries, coastal zone management (19% each), and coastal protection (32%). Additional thematic fields were mentioned by 20% of the participants and included underwater noise, acidification, invasive species, biodiversity, environmental economics, and social sciences.

## Results

### Inventory

A total of 42 DSTs were identified as being used in the Baltic Sea and drainage basin (Table 3). Assessments of the DSTs can be found in the DESTONY DST catalog^3^. Only 12 tools fulfilled all five DESTONY DST DC, and four of the DC were fulfilled by 14 tools. All 42 tools fulfilled the DC #3 (supports decision-making related to degradation of the aquatic environment). DC #2 (the tool can be accessed and operated on internet) turned out to be decisive as it was fulfilled by only ~40% of the tools and none of the tools with less than four fulfilled DC succeeded on this criterion. Most of the tools were directly available, i.e., found on the internet, but they usually lacked a web interface and required downloading and installation of a software or downloading a script or code to be run in e.g., R, Matlab or other software requiring licenses (e.g., GAMS). Approximately 25% of the DSTs were not directly available for end users, but only accessible through contact with the host. The criterion on interactivity, i.e., that the end user can modify inputs and/or settings of the tool, was fulfilled for about 80% of the tools. Examples of DSTs not fulfilling this criterion were tools in which the host feed data into a model and the end user only inspects the model outputs for a given area and/or time period, i.e., the required input from the end user is only to choose an area and/or a time period relevant for the specific problem setting.

**Table 3:** Identified decision-support tools (DSTs) listed in alphabetic order

Name	Category	Problem class	DAPSIWRM component^a^	Reference
**ACC-HUMAN**	Model	Contaminants	P, S, IW	Oltmans et al. (2019)
BALTCOST	Model	Eutrophication	IW, RM	Hasler et al. (2014)
**Baltic Explorer**	Planning tool	Sea-area use	D, A, P, S, IW, RM	BONUS BASMATI Project (2020)
BALTSEM-POP	Model	Contaminants	P, S	Undeman et al. (2015)
**BEAT 3.0**	Assessment tool	Biodiversity and conservation	S	Nygård et al. (2018)
**BIAS**	Model	Noise	P, S	Fyhr and Nikolopoulos (2016)
BSII	Assessment tool	Cumulative effects	A, P, S	Korpinen et al. (2013)
BSPI	Assessment tool	Cumulative effects	A, P	Korpinen et al. (2012)
**BWMC tool**	Assessment tool	Nonindigenous species	A, P	Ruiz and Sethuraman (2015)
**CHASE**	Assessment tool	Contaminants	S	Andersen et al. (2016)
**EcoImpactMapper**	Assessment tool	Cumulative effects	A, P, S	Stock (2016)
ERGOM-MOM	Model	Eutrophication	P, S	Neumann et al. (2002)
**EUTRO-OPER**	Assessment tool	Eutrophication	S	HELCOM (2015)
FIT	Assessment tool	Fishery management	A, P, S	Eigaard et al. (2016)
GETM-GITM	Model	Hydrography	S	Burchard and Bolding (2002)
**HEAT 3.0**	Assessment tool	Eutrophication	S	Fleming-Lehtinen et al. (2015)
**InSAT**	Assessment tool	Impact evaluation	S, IW, RM	Karnauskaitė et al. (2018)
InVest	Model	Impact evaluation	S, IW	Sharp et al. (2020)
**LPI**	Assessment tool	Biodiversity and conservation	P, S	Loh et al. (2005)
**MareFrame**	Stakeholder tool	Fishery management	A, P, S	MareFrame project (2020)
**Marmoni tool**	Assessment tool	Biodiversity and conservation	S	MARMONI project (2020)
**Marxan**	Planning tool	Sea-area use	A, P, S, IW, RM	https://marxansolutions.org/
**MESAT**	Assessment tool	Impact evaluation	S, I	Inácio et al. (2018)
**MIRACLE**	Stakeholder tool	Eutrophication	A, P	Neset and Wilk (2018)
MIRADI	Stakeholder tool	Biodiversity and conservation	A, P, S, IW, RM	https://www.miradi.org/
MONERIS	Model	Eutrophication	P	Venohr et al. (2011)
**Mytilus**	Assessment tool	Cumulative effects	A, P, S	Hansen (2019)
**NEAT**	Assessment tool	Biodiversity and conservation	S	Berg et al. (2019)
**NEST**	Model	Eutrophication	A, P, S, IW, RM	Wulff et al. (2013)
**POPCYCLING-Baltic**	Model	Contaminants	P, S	Wania et al. (2000)
RAUMIS	Model	Impact evaluation	A, P, S, RM	Kreins et al. (2007)
Recreation Site Values	Model	Impact evaluation	A, S, IW	Czajkowski et al. (2018)
SAF	Stakeholder tool	Impact evaluation	D, A, P, S, IW, RM	Støttrup et al. (2019)
SOCOPSE	Planning tool	Contaminants	P, S, IW, RM	Baartmans et al. (2009)
**Stakeholder Preference and Planning Tool**	Stakeholder tool	Impact evaluation	IW, RM	Schumacher et al. (2018)
Symphony	Model	Sea-area use	A, P, S	Swedish Agency for Marine and Water Management (2017)
TargetEconN	Model	Eutrophication	IW, RM	Hasler et al. (2019)
**Tool4MSP**	Planning tool	Sea-area use	A, P, S, IW	Menegon et al. (2018)
**WATERS IA tool**	Assessment tool	Eutrophication	S	Lindegarth et al. (2016)
**VEMALA**	Model	Eutrophication	A, P, S	Huttunen et al. (2016)
**VEMU 3**	Assessment tool	Eutrophication	P, S	Aroviita et al. (2019)
**Zonation**	Model	Biodiversity and conservation	D, A, P, S, IW, RM	Moilanen et al. (2014)

DSTs marked with bold font fulfilled 4 or 5 of the DST definition criteria (DC)

^a^*D* drivers, *A* human activities, *P* pressures, *S* state changes, *IW* impacts (on welfare), *RM* responses (management measures)

**Fig. 1:**
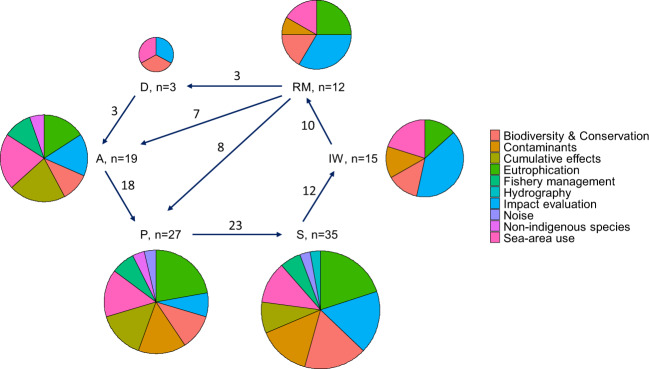
The representation of DSTs addressing different problem topics in the different DAPSIWRM framework segments. The DAPSIWRM framework and the links between the segments are based on Elliott et al. (2017). The pie chart area is scaled according to the number of DSTs (also indicated by *n*) and numbers on the arrows indicate number of DSTs linking the segments. A single DST can address several segments. D drivers, A human activities, P pressures, S state changes, IW impacts on welfare, RM responses (management measures)

**Fig. 2:**
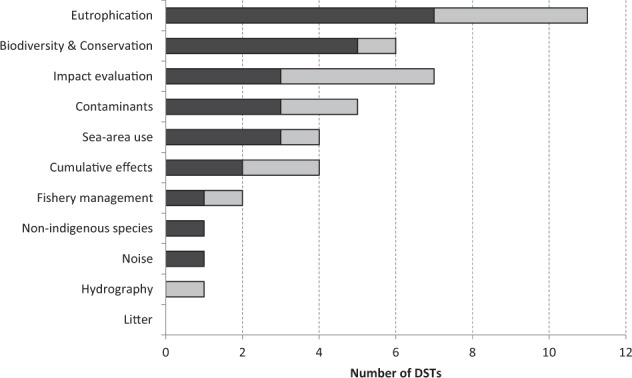
The distribution of tools among different environmental problem areas. The problem areas are ordered according to how many DSTs fulfilled at least four of the DST definition criteria (dark gray bars) and secondly according to number of tools fulfilling 1–3 of the definition criteria (light gray bars)

DSTs were found to respond to all segments of the DAPSIWRM framework and covered a broad range of environmental problems (Figs 1 and 2). Nine tools were specific to only one segment in the framework; of these, all but one treated state changes. Tools covering several segments often focused on the links between activities, pressures, and state changes. Drivers were only addressed by three DSTs and these were also the only ones to cover the whole DAPSIWRM framework. The three DSTs covering the full framework were of different types (model, stakeholder tool, and planning tool) and addressed different issues (biodiversity and conservation, impact evaluation, and sea-area use). All DAPSIWRM segments, apart from drivers, could be covered using a single DST for problems related to eutrophication, whereas for problems related to contaminants, several tools were needed to cover the same segments. No tools dealing with cumulative effects of pressures, fishery management, noise, nonindigenous species, or hydrography were found to address issues related to impacts on welfare or responses as measures.

Most of the DSTs were different types of assessment tools (17 tools) and models (16 tools), but also planning tools (4 tools) and tools for stakeholders (5 tools) were identified. Both models and assessment tools covered a wide range of environmental problems and could be used to assess environmental status or pressures. Also societal impacts and measures, including cost-effectiveness, could be evaluated with these tools. Planning tools mainly addressed sea-area use, i.e., MSP. Stakeholder tools, i.e., the tool type for interactions with stakeholders, covered a variety of problem settings including fisheries, eutrophication, conservation, and impact evaluation.

### DST Performance

**Fig. 3:**
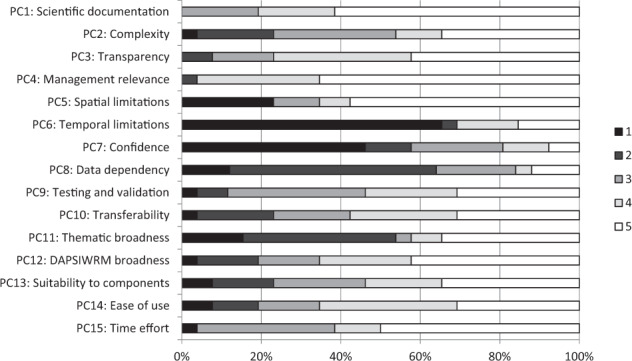
Proportions of tool scorings according to the performance criteria. *N* = 26 (number of tools evaluated = 26). Full descriptopns of the performance criteria (PC) and the scoring classes (1–5) are presented in Table 2

DSTs fulfilling at least four of the DC were analyzed in more detail regarding their performance (DC listed in Table 1, PC scoring described in Table 2 and illustrated in Fig. 3). A clear majority of these DSTs scored high (5 or 4) in PC4 management relevance to the Baltic Sea (96% of the DSTs), PC1 scientifically documented (81% of the DSTs), and PC3 transparency (77% of the DSTs). In contrast, the DSTs scored low (1 or 2) in PC6 temporal limitations (69% of the DSTs), PC8 data dependencies (62% of the DSTs), and PC7 confidence (58% of the DSTs).

**Fig. 4:**
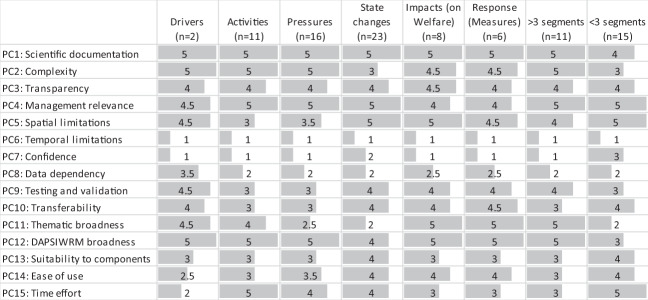
Median scores of decision-support tools according to the segments of the DAPSIWRM framework they address. The length of the gray bars correspond to the median score. Performance criteria (PC) and scoring classes (1–5) are presented in Table 2. Note that the same tool can address several segments. *N* = 26

Some general pattern could be observed regarding how the DSTs covered the DAPSIWRM framework (Fig. 4). DSTs covering less than three DAPSIWRM segments, generally had higher scores in PC7 confidence and PC15 time effort, than tools covering more than three DAPSIWRM segments. Vice versa, DSTs covering more than three DAPSIWRM segments applied more complex methodologies (PC2) and were thematically broader (PC11) than DST that inspected the DAPSIWRM framework more narrowly. Tools inspecting activities (*n* = 11), pressures (*n* = 16), or state changes (*n* = 23) had high management relevance (PC4), but where typically sensitive to the type of input data (PC8) and were thematically narrower (PC11) than tools exploring the other DAPSIWRM segments. DSTs addressing impacts on welfare (*n* = 8) and responses using measures (*n* = 6) were generally thematically broad and flexible to different policy issues (PC11).

**Fig. 5:**
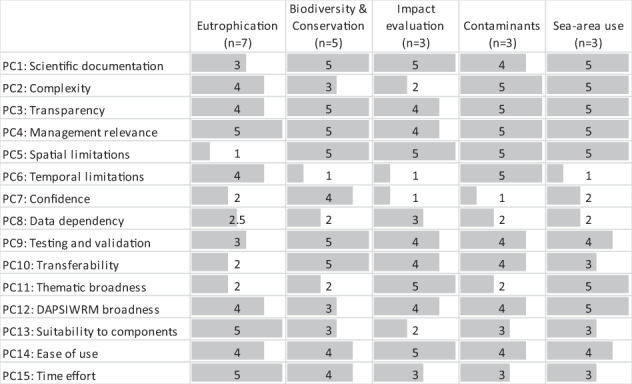
Median scores of decision-support tools according to the environmental problem area they address. The length of the gray bars correspond to the median score. Performance criteria (PC) and scoring classes (1–5) are presented in Table 2. *N* = 26 (results shown only for problem area with more than three DSTs)

Comparing DSTs developed for different problem areas, some general patterns could be observed, although the number of tools per problem area was low (Fig. 5). Opportunities to examine temporal changes (PC6) were best implemented in tools related to eutrophication (*n* = 7) and contaminant pollution (*n* = 3). DSTs addressing eutrophication were generally also well compatible with available input data and the outputs were operationally used (PC13). Confidence (PC7) was best documented in DSTs dealing with biodiversity issues (*n* = 5). Tools addressing impact evaluation (*n* = 3) and sea area use (*n* = 3) generally had a broad thematic scope (PC11), entailing several policy issues. Transparency (PC3) and management relevance (PC4) was generally high in DSTs in all problem areas.

**Fig. 6:**
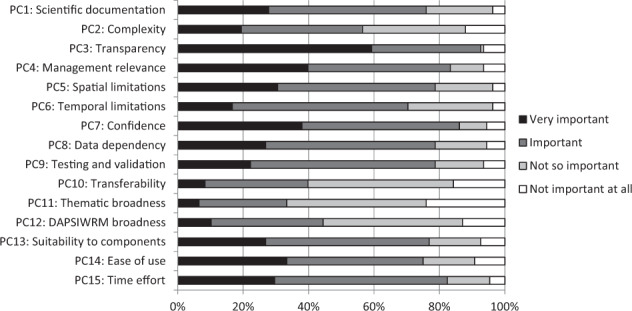
Proportions of answers from the end-user evaluations of the importance of the performance criteria. *N* = 108 (number of answers = 108). The full descriptions of the performance criteria (PC) are presented in Table 2

Through the eyes of an end user, the most important features of a DST included PC3 transparency, PC7 confidence, PC4 management relevance to the Baltic Sea, and PC15 time effort, which 93%, 86%, 83%, and 82% of end users, respectively, rated as very important or important (Fig. 6). PC15 Time effort was especially valued by end users working in administration. PC deemed unimportant by end users were PC11 thematic broadness, PC10 transferability, and PC12 DAPSIWRM component broadness, for which 67%, 60%, and 56% of end users, respectively, responded not important at all or not so important.

### Responses from Hosts on Tool Development

**Fig. 7:**
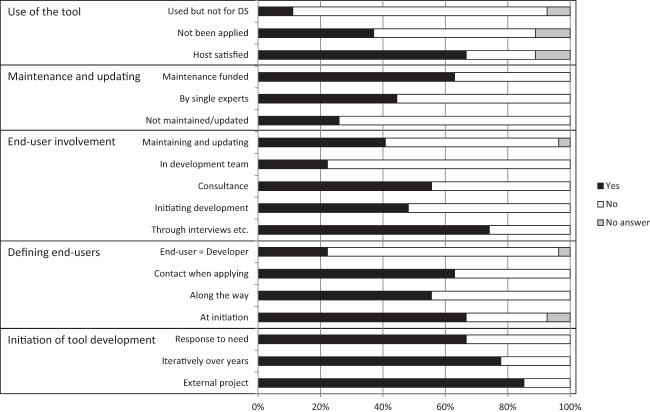
Results from the questionnaire to DST developers/hosts on the involvement of end users in the development of the tools. *N* = 27 (27 DST developers/host responded to the questionnaire). The full questions are presented in Online Resource 1

Approximately two thirds of the DST hosts answered the survey related to the development and current use of the tools (Fig. 7). According to the answers, two thirds of the tools were initiated as a response to a management need. End users had a strong role in the initial tool development process for half of the DSTs. Once developed, the DSTs are maintained and updated to a varying degree as 60% of the developed DSTs had allocated funding for maintaining and updating the tools. A quarter of the tools are not actively updated or maintained. More than a third of the tools are reported not to be used for environmental management as intended by the tool hosts/developers.

The involvement of end users in DST development varied between different problem areas (Table 4). Tools addressing eutrophication-related problems were normally developed in close cooperation with end users, and in half of the DSTs end users were part of the development team. DSTs concentrating on impact evaluations and cumulative effects also had good contact with end users. For tools addressing questions related to biodiversity and conservation as well as contaminants, end users were not well integrated into the development process. Although contact with end users was maintained when developing tools related to fishery management, nonindigenous species and underwater noise, the end users were not directly involved in the development teams.

**Table 4:** The number of DSTs in which end users have been involved in the development of the DSTs for different problem topics

	Eutrophication (*n* = 5)	Impact evaluation (*n* = 5)	Biodiversity and conservation (*n* = 4)	Contaminants (*n* = 3)	Cumulative effects (*n* = 4)	Sea-area use (*n* = 2)	Fishery management (*n* = 1)	Nonindigenous species (*n* = 1)	Noise (*n* = 1)	Hydrography (*n* = 1)
Through interviews etc.	5	5	2	1	2	1	1	1	1	1
Initiating development	3	1	2	1	3	0	1	1	0	1
Consultance	4	3	2	1	2	1	0	1	0	1
In development team	2	2	1	0	0	0	0	0	0	1
Maintaining and updating	3	2	3	1	0	1	0	0	0	1

Note that we did not get answers to from all developers/hosts, *N* = 27. See Online Resource 1 for full information on the questionnaire

To evaluate if (i) DSTs with end-user involvement in development, (ii) DSTs developed in response to a need, or (iii) DSTs where hosts were satisfied with the tool use perform better than where this has not been the case, we chose the three PC that end users valued the most, namely PC3 transparency, PC4 management relevance to the Baltic Sea, and PC7 confidence. However, only small differences were found in the median values between the groups.

## Discussion

The DSTs in our inventory often focused on specific environmental problems, and seldom provided solutions for a complete ecosystem approach or integrated management of marine environment. The problems addressed in the tools mirror the main challenges in the Baltic Sea (HELCOM 2018c; Reusch et al. 2018). The most addressed problem in the existing DSTs, eutrophication, as well as the frequently addressed biodiversity and contaminant issues are defined as priority areas in the HELCOM BSAP. Also, other marine environmental policies relevant for the Baltic Sea, such as the EU WFD and the MSFD, focus on these environmental issues. Tools addressing aspects relevant for the Maritime Spatial Planning Directive, e.g., trade-offs between human activities and conservation, were also well represented. Relatively new aspects from a marine management point-of-view, such as underwater noise and litter, were on the other hand incompletely represented in the inventory.

### Compliance with the Ecosystem Approch

One of our main interests was to investigate how the DSTs fit into the DAPSIWRM framework, as the ecosystem approach currently is the aim in marine management (Atkins et al. 2011; Elliott et al. 2017; Hyytiäinen et al. 2014; Reusch et al. 2018; Scharin et al. 2016). As DSTs most often are designed to deliver answers to specific question settings (Sullivan 2004), it was not expected that many tools would address the whole DAPSIWRM framework. This was also the case, as only three tools were found to consider all segments. Most often, three or less segments of the DAPSIWRM framework could be addressed with a single tool, with a dominant focus on the activities–pressures–state changes segments or links between them. DSTs were found to address this part of the DAPSIWRM framework in almost all considered environmental problem areas. The links between activities and pressures are well-defined (e.g., HELCOM 2018b), and DSTs focusing on single pressures and DSTs estimating cumulative effects were found in the inventory. Dynamic models assessing pressure-related environmental changes and tools mapping pressures spatially were also found. Some of these DSTs covered the entire Baltic Sea, while others were directed toward supporting management at nation level. DSTs estimating effects of cumulative pressures were based on similar principles and all had a spatial approach. State assessment tools were often indicator-based, providing systems for integration and aggregation of indicator data.

It is evident that tools addressing the impacts on welfare (the socio-economic perspective) and linking to management responses are clearly underrepresented compared to those concentrating on the environmental and ecological impacts of human activities and the pressures they cause. A relatively large number of cost-effectiveness models have been developed and used to assess nutrient abatement in the Baltic Sea (e.g., Ahlvik et al. 2014; Elofsson 2010; Hasler et al. 2014; Hasler et al. 2019; Hyytiäinen et al. 2014), but the use of these tools to support choices between policy options are relatively rare. Some models are used in cost–benefit analysis frameworks that could qualify as DSTs (Hyytiäinen et al. 2014; Scharin et al. 2016), but those made for the Baltic Sea have not been developed into DSTs. Also, Pınarbaşı et al. (2017) found that DSTs used in MSP mostly focused on environmental problems and seldom incorporated socio-economic aspects, which was also pointed out as a weakness by end users of MSP tools (Pınarbaşı et al. 2019).

As DSTs addressing the impacts on welfare–responses using measures–segments of the DAPSIWRM framework are so strongly underrepresented, we may conclude that the ecosystem approach is not very well expressed by the currently available DSTs. The fact that few tools addressing impacts on welfare were operational online, underpins the need for further development of this aspect. Mee et al. (2015) describe challenges of linking social and ecological components in the ecosystem approach, identifying an institutional gap between the disciplines and e.g., differences in interpretation of impacts, while Hasler et al. (2016) and Scharin et al. (2016) discuss problems linking marine data and economic models, but also suggest solutions. Bateman et al. (2013) have, among others, demonstrated that this is possible for terrestrial ecosystems, providing guidelines for how an ecosystem services mapping exercise can be made and used for decision support. Although the DAPSIWRM framework clarifies such epistemic discrepancies (Elliott et al. 2017), solutions for better linkage of environmental and socio-economic aspects are not yet mirrored in the available DSTs. Tools that did estimate the impact on welfare included a number of integrated indicator-based assessment methods, which were not exclusively focused on environmental aspects, but also considered economic aspects as well as contributions to social well-being or welfare. These included sustainability assessments methods, cost–benefit analysis tools, ecosystem services assessment methods, as well as tools that assess public or stakeholder concerns and preferences. DSTs evaluating responses and measures linked back to the other segments in the DAPSIWRM framework. Most frequently, measures were linked to state changes, but also activities, pressures, and impacts on welfare were often addressed, allowing for a more holistic evaluation of the effectivity of measures.

Drivers, setting the requirements for management, are formed by basic human needs (food, space, recreation, etc.), but few of the existing DSTs actually identified such drivers. Many of the DSTs in our inventory responded to specific question settings, where the societal drivers might be clear although not directly included in the tool. In comparison to the tools used in management, those directed toward citizens to raise awareness on the impact of their consumption, such as carbon footprint or nutrient pollution calculators (not included in the inventory), often take a starting point from the basic needs of people such as housing, food, and transportation. Citizens can, based on the drivers, evaluate how their consumption choices impact their welfare. Including clearer links to the drivers in management tools could help in identifying alternative solutions to fulfill the needs, thus facilitating the assessment of impacts on welfare. Consequently, this would facilitate appliance of the ecosystem approach.

### Performance of DSTs and End User Preferences

In general, the DSTs included in our inventory were well documented and transparent. DSTs are most often science-driven (Bolman et al. 2018), meaning that transparency and repeatability (i.e., that methodology is sufficiently described), is of high standard in the tools. Transparency was also, together with confidence, one of the most important properties end users pointed out. Transparency and confidence are important factors in the decision-making process, as managers may need to defend their decision choices to stakeholders (Verweij and van Densen 2010). Confidence estimates can facilitate the decision-making, whereas transparency makes it easier to explain the background and defend the decision choice.

Confidence was one of the PC scoring lowest among the tools in our study. Challenges in estimating the uncertainty is often a reason for not communicating uncertainty in DST outputs (Borja et al. 2016; van Beest et al. 2020). As measures can be expensive to carry out, managers want to be confident in any decision taken. In environmental management, reducing the uncertainty in available information can prove cost-efficient (Nygård et al. 2016). van Beest et al. (2020) discuss possibilities for improving communication of uncertainty in decision-support tools, arguing that in order to successfully manage the environment, sources of uncertainty need to be quantified, incorporated, and communicated throughout the tool development and decision-making process.

Broadness of tools, both in ability to inspect several policy issues and segments in the DAPSIWRM framework, was generally not considered important by end users. In fact, this was also reflected in the existing DSTs: they did not perform well in the criterion. As mentioned, DSTs should provide answers to specific questions (Sullivan 2004) and this is obviously also something end users value. The challenges in applying the ecosystem approach in a single DST discussed earlier in combination with the wish for specific DSTs imply that instead of one universal tool, a toolbox of DSTs seem to be the most realistic way to implement the ecosystem approach in marine management. An optimal way forward would be that the DSTs in this toolbox had the ability to utilize the outputs from one tool as inputs into the next one. Such a toolbox would maintain transparency, but potentially impede estimates of uncertainty sources to be retained throughout the management chain.

Although the spatial dimension is well incorporated in the DSTs, the examination of temporal aspects is usually restricted. The DSTs are most often temporally fixed, e.g., to the current situation or to a certain assessment period or scenario, not incorporating temporal trends or projections into future. A similar lack in DSTs used in MSP was also identified by Pınarbaşı et al. (2017). DSTs are usually also dependent on a certain type of data input, not always flexible to fully incorporate e.g., available monitoring data or operationally produced data products such as indicator data.

### Host Experiences

A marked number of the DST hosts were of the opinion that their tool had not been applied by the management community to the extent expected and/or could not say that they were satisfied with the way the end users applied their tool. Part of the hosts had the impression that their tool was applied broadly, although not for decision-making. Put together, this suggests that the existing DSTs are not applied to their full potential. Furthermore, it seems that even though many end users are well informed, there is an information gap between the supply/development and use/demand of DSTs.

One way of increasing the knowledge, experience, and commitment of end users to DSTs is through involvement already during the development phase. End user involvement in DST development is important for improving user-friendliness and ability to account for issues related to the decision-making process (Pınarbaşı et al. 2017; Bolman et al. 2018). End user involvement is also needed to improve inclusion of existing regulatory and legal demands (Pınarbaşı et al. 2019). We suggest that a common feature to a successful DST is early involvement of end users, since this promotes end user commitment and ensures a more accurate implementation of their needs. Most of the DSTs in the Baltic Sea area were developed in response to a need, and half of the DSTs were actually initiated by end users. End users, however, had been part of the development team in less than a quarter of the cases. Involvement through interviews and questionnaires was more common, but to secure that DSTs are user-friendly (also for non-scientists) and increase their use, closer involvement of end users is recommended.

## Concluding Remarks

In summary, the currently available DSTs address the major environmental problems in the Baltic Sea. To facilitate the implementation of the ecosystem approach, we suggest that attempts to combine the outputs of existing tools in further development of DSTs could lead to more holistic insight of the consequences of management actions. We recommend closer involvement of stakeholders and end users in the DST development phase, in order to improve the tool’s usability and thus, increase the value and impact of the DSTs in environmental management.

## Supplementary Information

Online Resource 1

Online Resource 2

## Data Availability

The collected material on the Decision-Support Tools is available in the DESTONY Database: http://nest.su.se/bonus_dst/.

## Footnotes

1 https://www.bonusportal.org/projects/synthesis_2018-2020/destony

2 https://www.bonusportal.org/projects

3 http://nest.su.se/bonus_dst/
