# Supplementary material for: Decision-Support Tools Used in the Baltic Sea Area: Performance and End-User Preferences
Source: Environ Manage. 2020 Sep 10;66(6):1024–38. doi: 10.1007/s00267-020-01356-8 (PMC7686007; doi:10.1007/s00267-020-01356-8)
Supplement: Supplementary file 1 — Online Resource 1 [file 267_2020_1356_MOESM1_ESM.docx]

Online Resource 1

Questionnaire sent to DST hosts/developers to gain understanding on development and end-user involvement, as well as use of the DSTs.

| Questions to DST host / developer |
| --- |
| **1) Initiation of tool development** |
| *What initiated the development of the tool?* |
| ○ It was developed as part of an externally funded project (yes/no) |
| ○ Technically it has been developed along the years, not through a single project (yes/no) |
| ○ It was developed as a response to a concrete need, for specific management use (yes/no) |
| **2) Defining end-users** |
| *Were the end-users clearly identified?* |
| ○ The end-users were identified already when initiating tool development (yes/no) |
| ○ Potential / actual end-users have been defined along the way (yes/no) |
| ○ End-users are in contact with the host when applying the tool (yes/no) |
| ○ It is hard to differentiate between end-user and tool developer / tool host (yes/no) |
| **3) End-user involvement** |
| *How are the end-users involved in developing and applying the tool?* |
| ○ They were involved in the development phase through interviews, questionnaires, workshops etc. (yes/no) |
| ○ They had a strong role in initiating the development of the tool (yes/no) |
| ○ They were actively participating in the development through consultancy (yes/no) |
| ○ They were actually in the tool development team, together with the experts (yes/no) |
| ○ They are involved in sustaining and updating the tool (yes/no) |
| **4) Maintaining and updating** |
| *How is the tool maintained and updated after the development phase?* |
| ○ It is not sustained/updated, due to lack of funding or other issues (yes/no) |
| ○ It is sustained by single experts personally (yes/no) |
| ○ Sustaining and updating is funded and taken care of even after the development phase (yes/no) |
| **5) Use of the tool** |
| *Are you satisfied with how the tool has been applied to support decision making?* |
| ○ We are happy in the way the tool is applied to support decision making (yes/no) |
| ○ The management community has not begun applying our tool to the extent we expected (yes/no) |
| ○ We believe the tool is used broadly, but actually not to support decision making (yes/no) |
