# Supplementary material for: Decision-Support Tools Used in the Baltic Sea Area: Performance and End-User Preferences
Source: Environ Manage. 2020 Sep 10;66(6):1024–38. doi: 10.1007/s00267-020-01356-8 (PMC7686007; doi:10.1007/s00267-020-01356-8)
Supplement: Supplementary file 2 — Online Resource 2 [file 267_2020_1356_MOESM2_ESM.pdf]

Experience of Decision Support Tools (DSTs)

This questionnaire is created as a part of the project BONUS DESTONY. The aim of BONUS DESTONY is to identify Decision Support Tools (DSTs) developed to assist ecosystem-based management of the Baltic Sea and its drainage basin and analyze them against the current and future needs. One of the outcomes of the project is an internet-based catalogue where all identified DSTs are evaluated according to a set of quality critera.

What is a Decision Support Tool?

Our definition is taken broadly, including for instance quantitative assessment tools, model-based management tools and operational systems linked to databases – the criteria being that they are applied to support decision-making when aiming at a sustainable marine environment in the Baltic Sea.

BONUS DESTONY receives funds from BONUS (Art. 185), which is jointly funded by the EU, the Academy of Finland and the Swedish Research Council Formas. Read more about the project here: https://www.bonusportal.org/projects/synthesis_%282018-2020%29/destony

What country do you work in? *

- ☐ Finland
- ☐ Denmark
- ☐ Estland
- ☐ Germany
- ☐ Latvia
- ☐ Lithuania
- ☐ Poland
- ☐ Russia
- ☐ Sverige
- ☐ ________

What kind of organisation do you work for? *

- ☐ Local administration
- ☐ Regional administration
- ☐ National administration
- ☐ International administration
- ☐ Non-governmental organisation (NGO)
- ☐ Research institute/University
- ☐ ________

Which organisation do you work for (optional)?

What field/fields do you work in? *

- ☐ Eutrophication
- ☐ Marine litter
- ☐ Fisheries
- ☐ Hazardous substances
- ☐ Marine habitats
- ☐ Marine spatial planning
- ☐ Coastal protection
- ☐ Nature protection
- ☐ Integrated Coastal Zone Management
- ☐

Is your work connected to any of these policies?

- ☐ Baltic Sea Action Plan
- ☐ Biodiversity Strategy
- ☐ Birds and Habitat Directives
- ☐ Blue Growth Strategy
- ☐ Common Agriculture Policy
- ☐ Common Fisheries Policy
- ☐ Floods Directive
- ☐ Marine Strategy Framework Directive
- ☐ Maritime Spatial Planning Directive
- ☐ Water Framework Directive
- ☐

How do you grade your experience with DSTs (Decision Support Tools)? *

- ☐ I don't know what it is
- ☐ I have an idea of what they are for
- ☐ I have used the outcome of the tools (for example processed data)
- ☐ I am a primary user (run the database/software)
- ☐ I have taken part in the development of them
- ☐

For what purpose/purposes do you use DSTs?

- ☐ To get a first idea of things
- ☐ To narrow down uncertainties

☐ For communication with stakeholders and public
☐ To overcome data gaps
☐ To assess different scenarios
☐ To assess a specific problem
☐ As input for own/other assessments/analysis
☐

Which of the following aspects has stopped you from using DSTs?

☐ Time constraints
☐ Financial constraints
☐ Lack of experience
☐ Lack of data
☐ Lack of knowledge about availability of tools
☐ Lack of DSTs for my area of work
☐ Lack of DSTs for my regional spatial scale
☐ Lack of acceptance by stakeholders/public
☐

How familiar are you with the following DSTs and how do they live up to your expectations? Please mark one of the options for each tool.

	I don't know it	I have heard of it	I have used it directly or indirectly and I am overall satisfied	I have used it directly or indirectly and I am overall unsatisfied
ACC-HUMAN (A Food Chain Model to Predict Bioaccumulation of Persistent Organic Contaminants in Humans from Environmental Levels)	○	○	○	○
Baltic Explorer (a web-based spatial decision support system for marine spatial planning)	○	○	○	○
Baltic Nest: Atmospheric data	○	○	○	○
Baltic Nest: Marine distributed databases	○	○	○	○
Baltic Nest: SANBALTS	○	○	○	○
Baltic Nest: BALTSEM	○	○	○	○
Baltic Nest: Fish modelling results (EwE)	○	○	○	○
Baltic Nest: Cost minimisation model	○	○	○	○

Baltic Nest: Riverine database	○	○	○	○
Baltic Nest: Catchment data	○	○	○	○
BALTSEM-C	○	○	○	○
BALTSEM-POP	○	○	○	○
BEAT 3.0 (Biodiversity Assessment Tool)	○	○	○	○
BIAS (Baltic Sea Information on the Acoustic Soundscape)	○	○	○	○
BONUS MIRACLE tool	○	○	○	○
BWMC Tool (Risk Assessment Tool under the HELCOM/OSPAR Harmonised Procedure on Exemptions under the Ballast Water Management Convention)	○	○	○	○
BSII (Baltic Sea Impact Index)	○	○	○	○
BSPI (Baltic Sea Pressure Index)	○	○	○	○
CHASE (Chemical Status Assessment)	○	○	○	○
DAS (Data Assimilation System)	○	○	○	○
EcoImpactMapper (a tool for mapping human impacts on marine ecosystems)	○	○	○	○
ERGOM (Ecological Regional Ocean Model)	○	○	○	○
EUTRO-OPER (Eutrophication Assessment Data and Information Workflow)	○	○	○	○
FIT (Fisheries Impact Tool)				
HEAT 3.0 (HELCOM Eutrophication Assessment Tool v3)	○	○	○	○
Indicator-based ICZM 'Best-practice' Evaluation Tool	○	○	○	○
InVest (Integrated Valuation of Ecosystem Services and Tradeoffs)	○	○	○	○
LPI (Living Planet Index)	○	○	○	○
MareFrame Decision Support Framework	○	○	○	○
Marmoni Biodiversity Assessment Tool	○	○	○	○

Marxan (a tool for marine spatial planning)	○	○	○	○
MEREK (Estonian national MSFD assessment tool)	○	○	○	○
MESAT (Marine Ecosystem Service Assessment Tool)	○	○	○	○
MIRADI Opens Standards	○	○	○	○
MONERIS (MOdelling NutrientEmissions in River Systems)	○	○	○	○
MoRe (Modeling of Regionalized Emissions)	○	○	○	○
NEAT (Nested Environmental Assessment Tool)	○	○	○	○
ODEMM (Options for Delivering Ecosystem-Based Marine Management)	○	○	○	○
POPCYCLING-Baltic	○	○	○	○
SOCOPSE (Source Control of Priority Substances in Europe)	○	○	○	○
Stakeholder Preference and Planning Tool	○	○	○	○
Symphony (a tool for ecosystem based marine spatial planning)	○	○	○	○
System Approach Framework (a guiding tool for a stepwise implementation of integrated coastal management)	○	○	○	○
VEMALA (a water quality and nutrient load model system for Finnish watersheds)	○	○	○	○
VEMU 3 (a tool for WFD status assessments in Finland)	○	○	○	○
WATERS Integrated Assessment Tool	○	○	○	○
Zonation (a tool for conservation planning)	○	○	○	○

If you are aware of any other DSTs, please provide the name and a brief description of the purpose for which you use it here.

What benefits have you experienced when applying DSTs?

Please write the name of the tool you are referring to in brackets.

What shortcomings or difficulties have you experienced when applying DSTs?

Please write the name of the tool you are referring to in brackets.

How important are the following quality criteria when you choose to use a DST? Please mark one of the options for each criterion.

	Not important at all	Not so important	Important	Very important
PC1: Scientific documentation *(Has the DST been documented in scientific publications?)*	○	○	○	○
PC2: Complexity of method *(How simple or complex is the method used for calculating the output?)*	○	○	○	○
PC3: Transparency of the DST *(Are all the processing described? Is the code public, the documentation understandable?) Are the underlying methods/calculations transparent for the user?*	○	○	○	○
PC4: Management relevance for the Baltic Sea *(To what extent is the output related to*				

making decisions on responses/measures?)

PC5: Spatial limitations *(Is the spatial scale of the tool restricted or can it be adapted according to management needs (e.g. applied on a local as well as national level)?)*

PC6: Temporal limitations *(Is the tool dynamic, i. e. describing changes over time?)*

PC7: Confidence assessment of results/Level of uncertainty *(Does the tool assess the uncertainty associated with the output and does this assessment account for all or a subset if potential uncertainties?)*

PC8: Data dependencies *(Does the tool work with missing values? Is it sensitive to changes in the type of input? Quantitative/qualitative data?)*

PC9: Testing and validation *(Has the DST been applied to different systems and tested independently?)*

PC10: Transferability *(How easily can the tool be adapted to other systems e. g. North Sea, fresh water systems?)*

PC11: Thematic broadness *(How generic is the DST? For example, which and how many policy issues (e.g. eutrophication, pollution, maritime activities) does it address?)*

PC12: Broadness of the DPSIR/DAPSIWRM addressed *(How broadly does the tool handle the management chain of events, from drivers to pressures, state changes, impacts to environment, social impacts and responses of society? E. g how many components in the DPSIR/DAPSIWRM cycle does it address?)*

PC13: Suitability to components operationally applied in the Baltic Sea *(How well does the tool fit in with the approaches and methodology already agreed upon in the area? Are the existing operational components (e.g. monitoring data, indicators) compatible with the tool when applied in the Baltic Sea? Is the output directly suitable as input or collaborative interpretation with outputs from other operational tools?)*

PC14: Ease of use/Expertise required *(Is the tool generally applicable to non-expert users or restricted to experts? Is the DST easy to apply? Is there need for expertise in a specific field, e.g. marine ecology, economics, policy?)*

PC15: Time effort *(How much time is needed to apply the DST? I. e. how much time is needed from the choice of the tool for a specific problem to the output of concrete/usable results?)*

Are there any other quality criteria that you consider important?

Do you see a lack of DSTs for a specific purpose or field? *

☐ Yes
☐ No

For which purpose or field?

Are you willing to develop your thoughts on DSTs in a face-to-face or Skype interview? *

☐ Yes
☐ No

Are you interested in receiving information about the results of this survey? *

☐ Yes
☐ No

Namn

First name Surname

Organisation

Email address

example@example.com

Skicka
